# Intra-renal microcirculatory alterations on non-traumatic hemorrhagic shock induced acute kidney injury in pigs

**DOI:** 10.1007/s10877-023-00978-7

**Published:** 2023-02-06

**Authors:** Bülent Ergin, Tom van Rooij, Alex Lima, Yasin Ince, Patricia AC Specht, Bert Mik, Ugur Aksu, Berna Yavuz-Aksu, Klazina Kooiman, Nico de Jong, Can Ince

**Affiliations:** 1 Department of Adult Intensive Care, Erasmus MC, University Medical Center Rotterdam, Erasmus University, Doctor Molewaterplein 40, 3015 GD Rotterdam, The Netherlands; 2 Department of Biomedical Engineering, Thorax Center, Erasmus MC, Rotterdam, The Netherlands; 3 Department of Translational Physiology, Academic Medical Center, Amsterdam, The Netherlands; 4 Laboratory of Experimental Anesthesiology, Department of Anesthesiology, Erasmus MC, Rotterdam, The Netherlands; 5 Department of Biology, Zoology Division, University of Istanbul, Istanbul, Turkey; 6 Duzen Laboratory Group, Biochemistry section, Istanbul, Turkey; 7 Laboratory of Acoustical Wavefield Imaging, Department of Applied Sciences, Delft University of Technology, Delft, The Netherlands

**Keywords:** Hemorrhagic shock, Resuscitation, Renal perfusion, Damage

## Abstract

Acute kidney injury (AKI) is frequently seen in patients with hemorrhagic shock due to hypotension, tissue hypoxia, and inflammation despite adequate resuscitation. There is a lack of information concerning the alteration of renal microcirculation and perfusion during shock and resuscitation. The aim of this study was to investigate the possible role of renal microcirculatory alterations on development of renal dysfunction in a pig model of non-traumatic hemorrhagic shock (HS) induced AKI.

Fully instrumented female pigs were divided into the two groups as Control (n = 6) and HS (n = 11). HS was achieved by withdrawing blood until mean arterial pressure (MAP) reached around 50 mmHg. After an hour cessation period, fluid resuscitation with balanced crystalloid was started for the duration of 1 h. The systemic and renal hemodynamics, renal microcirculatory perfusion (contrast-enhanced ultrasound (CEUS)) and the sublingual microcirculation were measured.

CEUS peak enhancement was significantly increased in HS during shock, early-, and late resuscitation indicating perfusion defects in the renal cortex (p < 0.05 vs. baseline, BL) despite a stable renal blood flow (RBF) and urine output. Following normalization of systemic hemodynamics, we observed persistent hypoxia (high lactate) and high red blood cell (RBC) velocity just after initiation of resuscitation resulting in further endothelial and renal damage as shown by increased plasma sialic acid (p < 0.05 vs. BL) and NGAL levels. We also showed that total vessel density (TVD) and functional capillary density (FCD) were depleted during resuscitation (p < 0.05).

In this study, we showed that the correction of systemic hemodynamic variables may not be accompanied with the improvement of renal cortical perfusion, intra-renal blood volume and renal damage following fluid resuscitation. We suggest that the measurement of renal injury biomarkers, systemic and renal microcirculation can be used for guiding to the optimization of fluid therapies.

## Introduction

Acute kidney injury (AKI) is an abrupt and usually reversible decline in renal function, typically within hours or days [1]. This is a frequent complication in patients admitted to the intensive care unit (ICU) and is associated with adverse outcomes including increased length of ICU and hospital stay, development of chronic kidney disease, and high short- and long-term mortality risk [2]. One of the main causes of AKI is hemorrhagic shock (HS) caused by excessive blood loss and persistent hypotension leading to a significant reduction of tissue and organ perfusion in trauma patients. Following this excessive blood loss, both macro- and microcirculation are compromised, resulting in tissue perfusion defects, hypoxia, production of reactive oxygen species (ROS), and inflammation [3, 4], finally resulting in multiple organ failure [5]. Amongst the failing organs, the kidneys are one of the most vulnerable organs suffering of hypoxic tubulars and endothelial injury leading to acute kidney injury (AKI), which is strongly associated with morbidity and mortality in critical illness [6]. Despite HS may cause a reduction of renal oxygen delivery by a diminished renal blood flow and perfusion, the systemic and renal autoregulatory systems are able to compensate the blood flow, perfusion, and oxygen delivery in short time after blood loss in organs. It has been documented that the hypovolemic status may further increase renal hypoxia due to intra-renal vasoconstriction induced by stimulation of sympathetic activity and activation of the renin-angiotensin system [7, 8].

Fluid resuscitation following HS is an important therapeutic approach in order to improve intravascular volume and organ perfusion in critical injured patients. However, based on their composition and volume, it has been reported that fluids may also have detrimental consequences on renal function [7] and renal microcirculation. Moreover, our previous experimental studies have revealed that renal microvascular PO_2_ level is also profoundly depleted [9, 10] and it is compromised much earlier than the other organs, such as the gut and heart [11]. It is now well-known that fluid resuscitation with balanced or unbalanced fluid, or hypertonic saline, did not correct the dysfunction of renal microvascular oxygenation, inflammation, and oxidative stress associated with HS despite a normalized blood pressure [10, 12]. Herein, we hypothesized that the alterations of intra-renal microcirculation may be a factor causing renal injury following hemorrhage and fluid resuscitation. However, the underlying mechanism of.

intra-renal microcirculatory alterations before and after fluid resuscitation is still unclear in AKI induced by non-traumatic hemorrhagic shock.

In this study, we investigated the following in a model of non-traumatic hemorrhagic shock and resuscitation: (1) the alterations of intra renal microcirculation by using Contrast-Enhanced Ultrasound (CEUS) during hemorrhagic shock and resuscitation, (2) the possible relationship between the systemic circulation, microcirculation, and oxygenation variables, and (3) whether or not there are parallel alterations between the sublingual and CEUS parameters after fluid resuscitation. We believe that the results of this study provide new insights on the intra-renal microcirculation, which leads to better understanding to guide renal therapy and prevent AKI.

## Materials and methods

Experiments were performed on 17 female pigs (crossbred Landrace × Yorkshire, 3–4 months old) with permission of the local animal experimental committee (EMC3379 142-14-01) of Erasmus University of Rotterdam, Erasmus Medical Center and in strict accordance with the principles of Declaration of Helsinki. Sample size was calculated according to a match paired analysis of expecting values of arterial pressure after resuscitation in HS group (mean diff:15, SD: 10, effect size: 1.5, a error: 0.05, power: 0.80, n = 6, 4 extra animal added for fall-out of HS group in case of losing the animal during HS and measurements failure (such as Swan-Ganz implementation, blood gas/electrolytes analysis, and sublingual microcirculation measurement). The animals were randomized into two groups: Hemorrhagic shock (HS group, n = 11) or Control (C group, n = 6). The mean ± standard deviation (SD) body weight of the animals was 28.6 ± 2.6 kg.

### Animal preparation

All animals were anesthetized by an intramuscular injection of mixture of tiletamine (5 mg/kg), zolazepam (5 mg/kg) (Zoletil, Virbac Laboratories, Carros, France) and xylazine (2.25 mg/kg) (Sedazine ® 2%, AST Farma B.V. Oudewater, the Netherlands). Anesthesia and fluid status were maintained with intravenous infusion of midazolam (1.5 mg/kg/h, Actavis, New Jersey, USA), ketamine (5 mg/kg/h, Alfasan, Woerden, the Netherlands), and sufentanil (4 µg/kg/h, Sufenta Forte, Janssen Pharmaceuticals Ltd. USA) in addition to 10 mL/kg/h Ringer Acetate (Sterofundin® ISO, B. Braun Melsungen, Germany) via the auricular vein throughout the experiments. Rocuronium bromide vas used for muscle relaxation (4 mg/kg/h, Fresenius Kabi, Germany). All animals were intubated through a midline cervical tracheostomy using an endotracheal tube (7.0 Fr). Animals were ventilated in a pressure-controlled mode (Servo 300, Siemens-Elema, Solna, Sweden) with a fraction of inspired oxygen of 0.40, a frequency that achieves normocapnia and a positive end-expiratory pressure of 5 cm H_2_O. Body temperature was monitored throughout experiments with a temperature probe in the nose and maintained at approximately 38–40 °C with a heating pad. The left femoral artery was cannulated with a 20-gauge catheter connected to a pressure transducer and used for sampling and continuous measurements of mean arterial blood pressure (MAP) and heart rate (HR). After catheterization of the right external jugular vein, which was also used to administer drugs and microbubbles injection, a Swan-Ganz catheter (Edwards Lifesciences, Irvine, CA, USA) was inserted via the introducer to measure central venous pressure (CVP), pulmonary arterial pressure (PAP) and cardiac output (CO). A midline abdominal incision was performed to insert a cystostomy tube into the bladder with double purse-string sutures for the collection of urine samples. To evaluate renal cortical perfusion using Laser Speckle Imaging (LSI) and intra renal microvascular perfusion by means of CEUS, the right kidney was exposed via an incision in the right flank. When all surgical procedures were completed, a perivascular ultrasonic transient time flow probe (Transonic Systems, Ithaca, NY, USA) was placed around the right renal artery and connected to a flow meter (model T206, Transonic Systems) for continuous measurement of renal blood flow (RBF).

### Experimental protocol and hemodynamic measurements

After completing the surgical procedures, a 45- to 60-minute equilibration period was allowed, and baseline measurements were performed after hemodynamic stabilization. After baseline measurements (BL, T0), blood was gradually withdrawn through a catheter in the right femoral artery until MAP reached a steady-state value < 50 mmHg and when lactate had increased > 3.0 mmol/L; this corresponded to ~ 40% of the total blood volume (total blood volume ≈ 8% of body weight, 38,7 ± 8,6 mL/kg blood withdrawn for induction of HS, mean±SD) and hemorrhage classification III or IV [13]. Following the simulation of severe hemorrhage, the animals were kept in this condition for 60 min to induce shock (T1) in HS group. The control group was fully instrumented, but did not undergo hemorrhage or fluid resuscitation. After this period, the catheter in the right femoral vein was used for fluid resuscitation with a balanced crystalloid (Sterofundin® ISO, B. Braun Melsungen, Germany) at dose of 50 ml/kg/h and measurements were performed 15 min. (early resuscitation, T2) and 60 min (late resuscitation, T3) after starting resuscitation. Hemodynamic parameters were monitored continuously during the entire experiment. Urine samples and arterial and venous blood, were taken at all-time points. At the end of the experiment, the animals were euthanized with a bolus of potassium chloride.

### Global oxygenation

Arterial oxygen content (CaO_2_) (ml O_2_/min) was calculated by the following equation; (1.34×[hemoglobin]×S_a_O_2_)+(0.003×P_a_O_2_). Central venous oxygen content (CvO_2_) (ml O_2_/min) was calculated as (1.34×[hemoglobin]×S_v_O_2_)+(0.003×P_v_O_2_). Global oxygen delivery was calculated as DO_2_ (L/min) = CO × CaO_2_. Global oxygen consumption was calculated as VO_2_ (L/min) = CO × (CaO_2_–CvO_2_).

### Blood gases, electrolytes, lactate, and creatinine

At every time point arterial blood samples (0.5 mL) were taken from the femoral artery and blood gas values, hemoglobin concentration, hemoglobin oxygen saturation, lactate, sodium, and potassium concentrations (ABL 800 flex blood gas analyzer: Radiometer, Copenhagen, Denmark) were determined immediately after blood withdrawal. Venous blood samples were also collected at each time point, centrifuged, and serum was stored at -80^o^C and thawed prior to analysis. Creatinine and urea (cobas 8000 c 502, Roche Diagnostics, Indianapolis, IN, USA) concentrations were both measured photometrically in serum.

### Renal function

Creatinine clearance (Clear_crea_ (mL/min)) was calculated with the following formula: Clear_crea_ = (*U*_*crea*_ × *V*)/*P*_*crea*_, where *U*_*crea*_ was the concentration of creatinine in urine, *V* is the urine volume per unit time and *P*_*crea*_ was the concentration of creatinine in serum. Serum neutrophil gelatinase-associated lipocaline (NGAL) levels were measured using the Enzyme-Linked Immunosorbent Assay (ELISA) kit (BioPorto Diagnostic, KIT 044). Serum sialic acid levels were analyzed according to Sydow’s method [14].

### Contrast-enhanced ultrasound imaging of the kidney

Contrast-enhanced ultrasound (CEUS) imaging was performed at all the time points. At each time point, a 1-mL bolus of Definity® contrast agent (Lantheus Medical Imaging, North Billerica, MA, USA) was manually injected into the jugular vein followed by a 10-mL saline. Measurements were recorded using a Vevo® 2100 preclinical high-resolution ultrasound scanner (FUJIFILM VisualSonics Inc., Toronto, Ontario, Canada) equipped with an MS250 transducer with the wide beam-width setting to ensure a low, more uniform transmit pressure over depth (18 MHz transmit frequency, 10 frames per second, 10% power, mechanical index 0.1). Immediately after injection of the contrast agent, ventilation of the animal was paused at an expiratory hold to minimize movement due to breathing. After 25–30 s, when the measurement was completed, ventilation was resumed. Cine loops of side-by-side B-mode and nonlinear contrast mode images were stored as lossless DICOM images for further offline analysis using MATLAB (The MathWorks, Natick, MA, USA).

### CEUS data analysis

The correction for tissue motion in the imaging plane was performed as described elsewhere [15, 16]. Briefly, the motion pattern of tissue in the field of view (FOV) was extracted from the B-mode images and applied to the contrast mode images to correct for motion in the FOV. Next, regions of interest (ROIs) were chosen for every DICOM recording, including an ROI in the deeper cortex for evaluating arterioles (> 8 mm depth) and two ROIs in the cortex from just below the exposed surface of the kidney to a depth of 1.5–2.5 mm for evaluating renal microcirculation. The intensities of all pixels in the renal artery and ROIs were calculated by the area of the ROI for every frame, resulting in a time-intensity curve (TIC) for each ROI.

Next, the semi-quantitative parameters of interest were obtained from these filtered TICs: peak-enhancement (PE) and full width at half maximum (FWHM) [17, 18]. In addition to these predefined parameters, we extracted another parameter which we previously defined as the intra-renal mean transit time (IRMTT) [19]; the arrival time of microbubbles flowing from the renal artery to the cortical microcirculation. No spatial differences were present in the data from two ROIs in the cortex, allowing for treating the data as one ROI. Our previous research also clearly showed that the two ROIs in the cortex were almost identical in both the control group as well as the experimental sepsis group [19].

### Laser speckle imaging (LSI) of the kidney

LSI was used to visualize the spatiotemporal changes of renal cortical perfusion during HS and in the control group at all time points, using the settings as described in our previous work [19]. Briefly, for LSI measurements, a commercially available system was used (Moor Instruments, Devon, UK), in which a near-infrared laser source operating at 785 nm illuminated the renal cortex with a penetration depth of approximately 1 mm [20].

Mean flux was analyzed and calculated as the mean ± SD of renal microvascular perfusion by using the Moor Instrument software.

### Sublingual microcirculation

Sublingual microcirculation was measured using the Cytocam (Braedius Medical, Naarden, the Netherlands), which is based on video microscopy and Incident Dark Field (IDF) technology [21]. To maximize the likelihood of a successful analysis of microvascular functionality, multiple videos were captured at each time point, varying the microscope’s focal depth by steps of 4 μm around the depth with the optimal focus (judged by the same experienced operator). All captured videos had a fixed pre-set duration of 100 frames (i.e., 4 s, given the 25 Hz acquisition rate offered by the device). The operator aimed to image the same area throughout the experiment; this was crucial to achieve an optimal match among the FOVs at the subsequent time points and to minimize the influence of anatomical spatial heterogeneity in the sublingual microcirculation on the functional parameters evaluated during video-analysis.

### Data analysis cytocam-IDF

After the quality assessment of videos, we selected 38 videos from 8 pigs of the HS group and 24 videos from 6 pigs of the control group. All those videos were automatically analyzed by using full-frame analysis of the MicroTools automatic software [22]. The microcirculatory parameters were described as total vessel density (TVD), functional capillary density (FCD), red blood cell velocity (RBCv), capillary hematocrit, and tissue red blood cell perfusion (tRBCp). Data are reported as percentage change of T3 with respect to BL with following formula [100*(T3 – BL)/BL].

### Statistical analysis

Data are expressed as the mean ± SD. Shapiro-Wilk tests were used for testing normal distribution of data. Repeated measures 2-way analysis of variance (ANOVA) (2 factors: time as a related sample factor and group as an independent sample factor) with *post hoc* Sidak’s and Tukey’s correction test for multiple analyses were used to determine inter- and/or intra-group differences of hemodynamic parameters, oxygenation, renal function, perfusion, biochemistry, CEUS derived parameters, and RBC velocity. An unpaired *t*-test was used for the analysis of the sublingual microcirculation. Statistical analysis was performed using GraphPad Prism version 7.0a for Mac (GraphPad Software, La Jolla, USA). A p-value < 0.05 was considered significant.

## Results

### Systemic hemodynamics

**Fig. 1:**
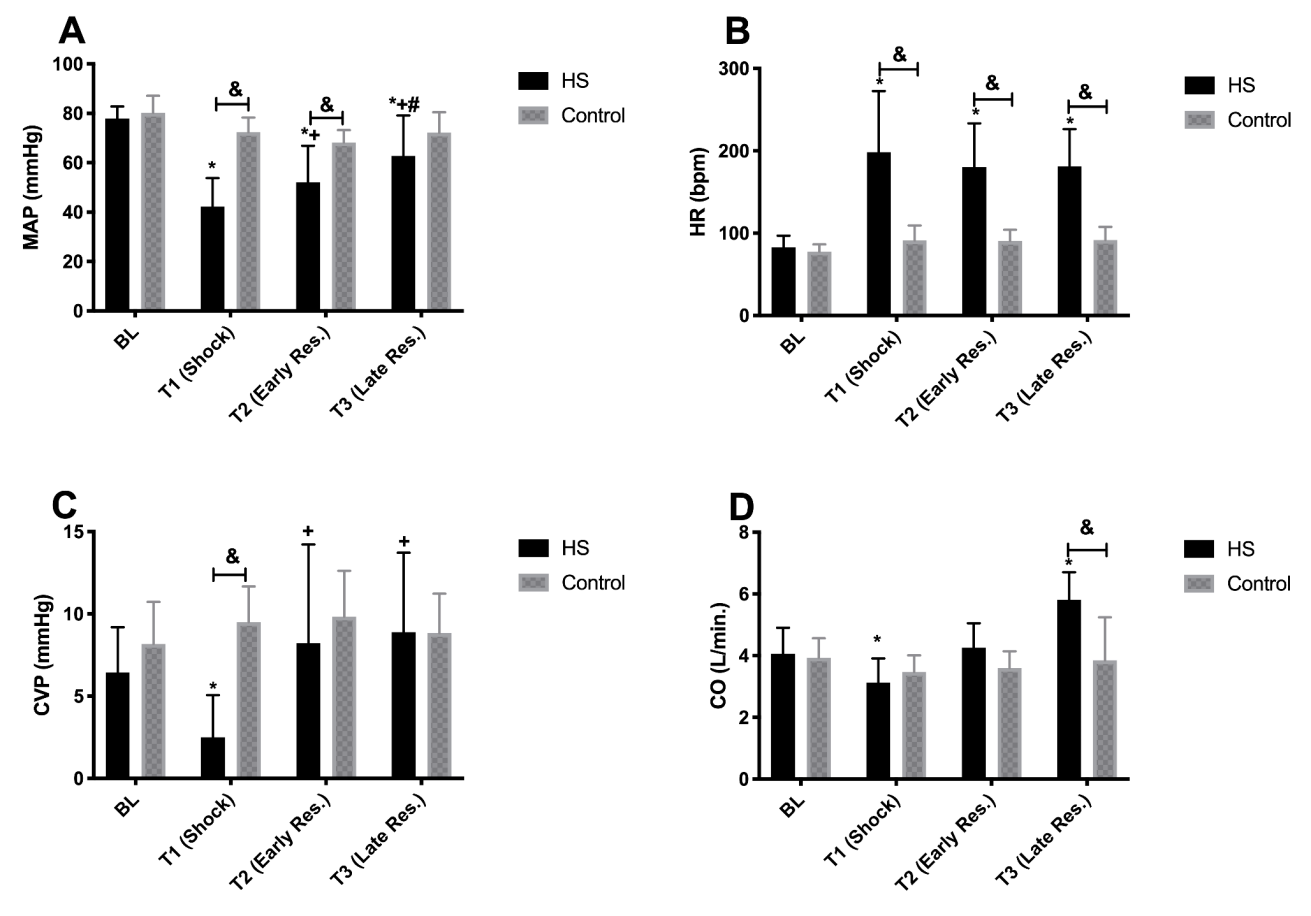
Mean arterial pressure (MAP), heart rate (HR), central venous pressure (CVP) and cardiac output (CO) throughout the experiment in the HS and Control groups. Values represented as mean ± SD: * p < 0.05 vs. BL; + p < 0.05 vs. T1; # p < 0.05 vs. T2; & p < 0.05 HS vs. Control

Data on the macro-hemodynamic variables are summarized in Fig. 1. Figure 1A shows that MAP in the HS group at T1, T2, and T3 was significantly different from BL measurements, drastically decreased during shock (T1), and gradually increased again during resuscitation (T2 and T3) to reach a level similar to that in the Control group at T3. In addition, MAP at T1 and T2 showed to be significantly lower than at the respective time points in the Control group. Figure 1B shows that HR was significantly higher at T1, T2, and T3 in the HS group than in the control group and HR significantly increased during shock (T1) and remained high during resuscitation (T2 and T3). Figure 1C shows that CVP dropped significantly during shock (T1) and improved again by resuscitation at T2 and T3 to levels comparable to BL. CO in the HS group decreased at T1 (shock), increased at T2 to a level comparable to BL, and considerably increased at T3.

PAP seemed to slightly, but not significantly, increase during the experiment in both the HS and Control group (Table 1). Only during late resuscitation (T3) PAP was significantly higher in the HS group than the Control group. SVR remained constant in the Control group but decreased in the HS group during the course of the experiment to ~ 50% of the starting value at BL and was significantly lower during late resuscitation (T3) than the control group at T3 (p < 0.05, Table 1).

**Table 1:** Pulmonary arterial pressure and systemic vascular resistance levels throughout the experiments in the HS and Control groups

	Hemorrhagic Shock	Control
Pulmonary arterial pressure (PAP, mmHg)		
BL	16.2±2.8	18.3±1.5
T1 (Shock)	17.4±3.7	20.6±2.5
T2 (early res.)	20.1±3.9	20.3±3.4
T3 (late res.)	24.5±5.8^*+#$^	19.1±3.6
Systemic vascular resistance (SVR, dyn.s.cm^− 5^)		
BL	1424±421	1424±270
T1 (Shock)	1141±292	1421±376
T2 (early res.)	798±162^*^	1219±186
T3 (late res.)	593±106^*+$^	1340±455

Values represented as mean ± SD * p < 0.05 vs. BL; + p < 0.05 vs. T1; #p < 0.05 vs. T2; & p < 0.05 HS vs. Control

### Clinical chemistry and hematology

All blood variables are summarized in Tables 2 and 3. No significant differences were observed in the etCO_2_ values throughout the experiment (Table 2). On the other hand, pH and base excess were lower after HS insult than at BL and in the Control group values, and they remained persistently lower during the entire resuscitation phase with respect to the Control (p < 0.05). Na^+^ and K^+^ concentrations remained constant in both groups during the entire experiment.

**Table 2:** Blood pH and electrolytes levels throughout the experiments in the HS and Control groups

	Hemorrhagic Shock	Control
pH		
BL	7.47±0.03	7.48±0.01
T1 (Shock)	7.35±0.07^*&^	7.47±0.04
T2 (early res.)	7.29±0.09^*+&^	7.45±0.04
T3 (late res.)	7.30±0.06^*&^	7.46±0.03
etCO_2_ (kPa)		
BL	5.6±0.5	5.5±0.1
T1 (Shock)	5.6±0.5	5.2±0.3
T2 (early res.)	5.7±1.1	5.0±0.1
T3 (late res.)	5.7±1.0	4.9±0.3
Base excess		
BL	3.6±2.1	3.8±1.8
T1 (Shock)	-4.5±4.4^*&^	2.9±2.3
T2 (early res.)	-6.6±4.0^*&^	2.3±2.6
T3 (late res.)	-5.3±4.8^*&^	2.1±3.0
Na^+^ (mmol/L)		
BL	140±1	143±1
T1 (Shock)	141±1	141±1
T2 (early res.)	142±2	142±2
T3 (late res.)	142±2	142±2
K^+^ (mmol/L)		
BL	3.9±0.3	3.8±0.2
T1 (Shock)	4.4±0.3	4.0±0.2
T2 (early res.)	3.9±0.4	3.9±0.3
T3 (late res.)	3.9±0.4	3.9±0.1

Values represented as mean ± SD * p < 0.05 vs. BL; + p < 0.05 vs. T1; & p < 0.05 HS vs. Control

**Table 3:** Hct, svO_2_%, plasma urea, urine osmolarity and plasma creatinine throughout the experiments in the HS and Control groups

	Hemorrhagic Shock	Control
Hct (%RBC)		
BL	24.9±1.6	22.7±2.1
T1 (Shock)	22.1±2.6^*&^	24.5±2.3
T2 (early res.)	17.7±2.9^*&^	24.3±2.2
T3 (late res.)	15.6±1.5^*&^	24.8±1.7
svO_2_%		
BL	66.7±9.9	62.6±10
T1 (Shock)	37.6±13.9^*&^	60.5±5.3
T2 (early res.)	49.5±11.4^*+^	61.3±5.6
T3 (late res.)	60.1±10.1^*^	67.2±8.0
Plasma Urea (mmol/L)		
BL	2.9±0.5	2.5±0.6
T1 (Shock)	5.0±0.8^*&^	3.4±0.8^*^
T2 (early res.)	4.8±0.8^*&^	3.5±0.8^*^
T3 (late res.)	4.7±0.7^*^	3.7±0.8^*^
Urine Osmolarity (mmol/L)		
BL	493±118	587±66
T1 (Shock)	512±164	679±107
T2 (early res.)	496±119	702±93
T3 (late res.)	411±97	640±265
Plasma Creatinine (µmol/L)		
BL	69.8±7.4	65.8±8.7
T1 (Shock)	114.6±30.2^*&^	70.5±5.9
T2 (early res.)	109.7±27.2^*&^	72.0±8.3
T3 (late res.)	104.0±28.9^*&^	72.5±6.7

Values represented as mean ± SD * p < 0.05 vs. BL; + p < 0.05 vs. T1; & p < 0.05 HS vs. Control

Table 3 shows that Hct levels reduced during HS and were further depleted in all the time points of resuscitation (p < 0.05) with respect to BL. Moreover, Hct remained low in all the time points of HS group in comparison to the same time points in the Control group. The svO_2_ level in the Control group did not changed throughout experiment. After induction of shock,  svO_2_ decreased significantly (p < 0.05), but normalized again to BL values after resuscitation (p < 0.05). Urine osmolality was stable in both groups. Urea levels in serum were higher in both the HS group and the Control group at T1, T2 and T3 in compared to BL values, but the plasma urea levels in HS group at T1 and T2 were further increased in comparison to the Control group). In addition, in the HS group urea concentration was higher during shock and early resuscitation than in the Control group. Urine osmolarity remained constant for both groups during the experiment. Serum creatinine levels were constant in the Control group but it increased upon induction of shock and remained high throughout the expriment. In addition, creatinine levels were almost 1.5 times higher than in the control group, which corresponds to AKI stage 1 according to KDIGO guideline [23].

### Global oxygen delivery and consumption

**Fig. 2:**
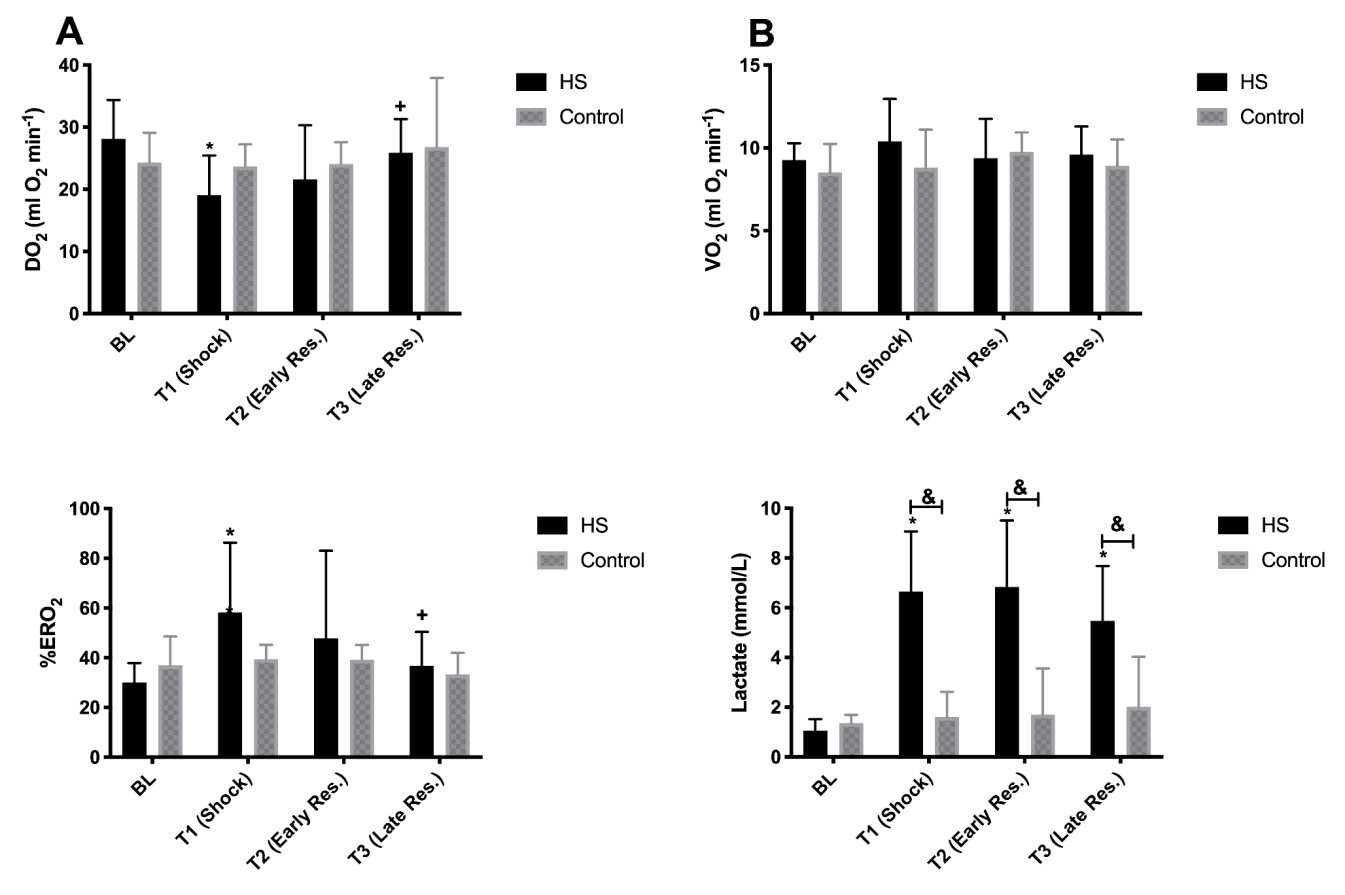
Systemic oxygen delivery (DO_2_), consumption (VO_2_), oxygen extraction rate (ERO_2_%) and plasma lactate levels throughout the experiments in the HS and Control groups. Values represented as mean ± SD * p < 0.05 vs. BL; + p < 0.05 vs. T1; & p < 0.05 HS vs. Control

Data on global oxygen delivery and consumption are summarized in Fig. 2. Systemic oxygen delivery (DO_2_) depleted during shock (T1) and early resuscitation (T2) and increased towards baseline during late resuscitation (T3). On the other hand, systemic oxygen consumption (VO_2_) was stable in both Control and HS groups throughout the experiments. Oxygen extraction rate (ERO_2_%) increased during shock (T1) and decreased again upon start of resuscitation (T2) and further decreased at late resuscitation (T3). Plasma lactate considerably increased throughout the experiment in the HS group, even after fluid resuscitation (T3), and was significantly higher than in the control group.

### Renal function, perfusion and damage

**Fig. 3:**
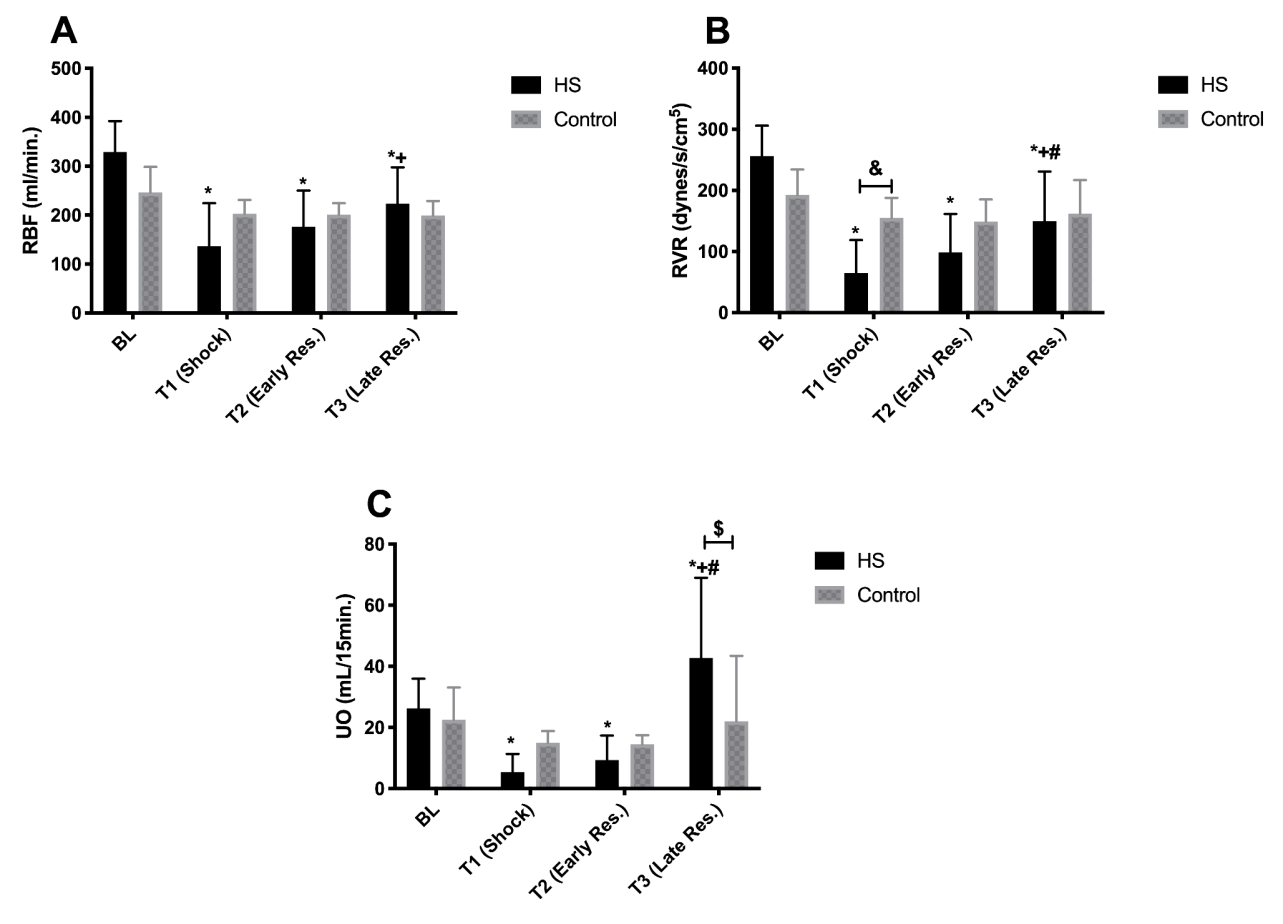
Renal blood flow (RBF), renal vascular resistance (RVR) and urine output (UO) throughout the experiments in the HS and Control groups. Values represented as mean ± SD * p < 0.05 vs. BL; + p < 0.05 vs. T1; # p < 0.05 vs. T2; & p < 0.05 HS vs. Control

**Fig. 4:**
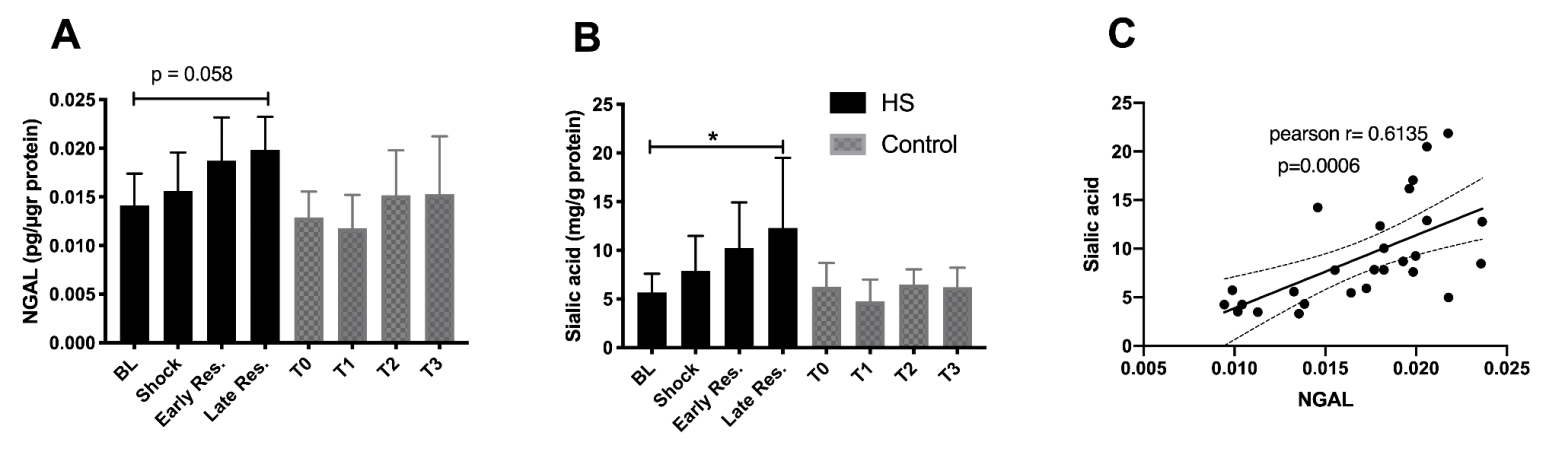
Serum neutrophil gelatinase-associated lipocalin (NGAL) and sialic acid levels throughout the experiments between the HS and Control groups (Panel A-B). Linear regression analysis between NGAL and Sialic acid (Panel C). Values represented as mean ± SD * p < 0.05 vs. BL.

The renal blood flow (RBF), renal vascular resistance (RVR) and urine output (UO) are represented in Fig. 3. RBF was lower in the HS group during shock and increased again during early and late resuscitation. RBF remained constant in the Control group. RVR was significantly decreased at T1 (shock) in comparison to BL and the Control group, and increased to levels comparable to the Control group during resuscitation. UO remained constant in the Control group but was lower at T1 and T2 than at BL in the HS group and significantly lower than at the respective time points in the Control group. After resuscitation, UO increased significantly due to the fluid load that was given and was also significantly higher than the Control group. Serum NGAL levels were normal in the HS group at T1 and T2, and marginally increased at T3 (p < 0.058 with respect to BL) (Fig. 4A). On the other hand, serum sialic acid levels increased during the experiment and were significantly higher at the end of the experiment than at BL in the HS group (p < 0.05). In the Control group, serum sialic acid levels remained constant over time (Fig. 4B). NGAL and sialic acid levels were correlated during shock and resuscitation (p = 0.0006) (Fig. 4C).

The creatinine clearance decreased after induction of shock and early resuscitation (p < 0.05 vs. T1 (shock)), at late resuscitation it was normalized again due to fluid resuscitation (p < 0.05 vs. T2 (early resuscitation)). In the Control group, no significant changes were measured in creatinine clearance (Table 4). The mean cortical perfusion, and perfusion heterogeneity values measured by LSI are presented in Table 4. Despite a stable perfusion heterogeneity in the HS group, the mean perfusion values reduced during shock at T1 (p < 0.05) and improved again after fluid resuscitation at T2 and T3 to BL values. Both the mean cortical perfusion and perfusion heterogeneity in the Control group were constant throughout the entire experiment.

**Table 4:** Mean Flux, perfusion heterogeneity and creatinine clearances levels throughout the experiments in the HS and Control groups

	Hemorrhagic Shock	Control
Mean Flux (A.U)		
BL	886±221	849±106
T1 (Shock)	667±227^*^	854±119
T2 (early res.)	790±126^+^	845±86
T3 (late res.)	821±120^+^	854±107
Perfusion heterogeneity (A.U)		
BL	0.23±0.02	0.2±0.01
T1 (Shock)	0.22±0.02	0.2±0.01
T2 (early res.)	0.23±0.03	0.2±0.01
T3 (late res.)	0.23±0.02	0.21±0.02
Creatinine Clearances (rational change from BL)		
BL	100±0	100±0
T1 (Shock)	28±17	110±54
T2 (early res.)	41±20^+^	127±62
T3 (late res.)	144±121^#^	128±91

Values represented as mean ± SD * p < 0.05 vs. BL; + p < 0.05 vs. T1; #p < 0.05 vs. T2/

### CEUS parameters

**Fig. 5:**
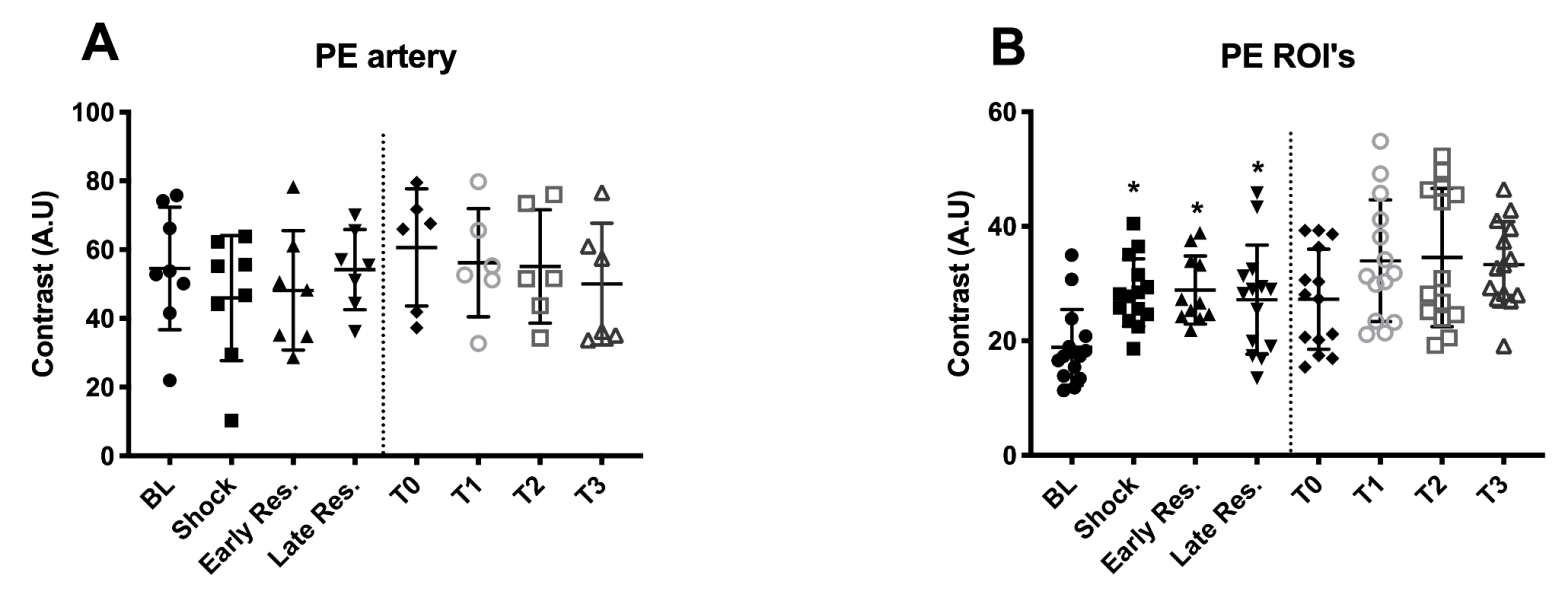
Peak enhancement (PE) in the arterial system and the cortical regions of interest (ROIs) throughout the experiments in the HS and Control groups. Values represented as mean ± SD * p < 0.05 vs. BL.

**Fig. 6:**
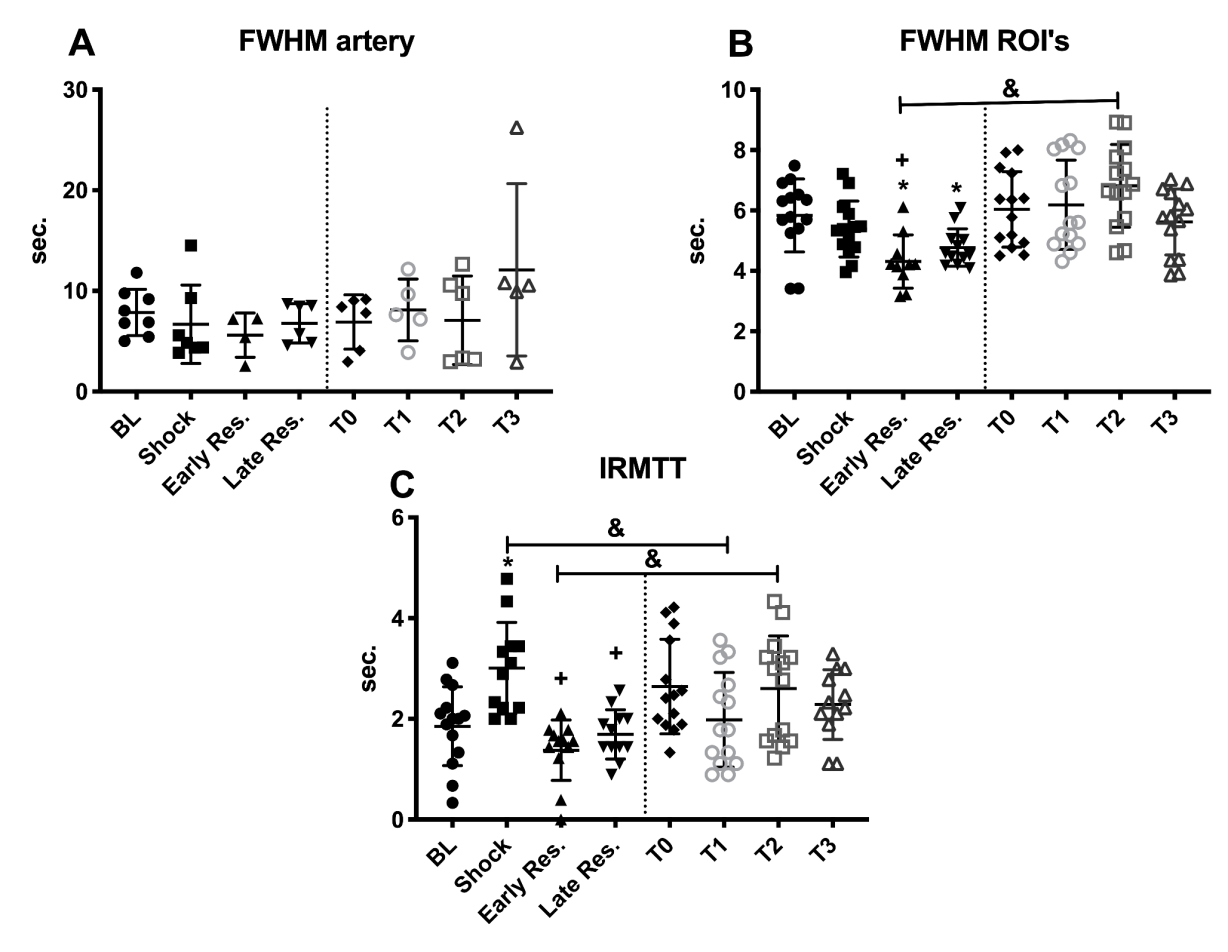
Full width at half maximum (FWHM) of the renal arterial system and the cortical regions of interest (ROIs), and the intra-renal mean transit time (IRMTT) throughout the experiments in the HS and Control groups. Values represented as mean ± SD * p < 0.05 vs. BL; + p < 0.05 vs. T1; # p < 0.05 vs. T2; & p < 0.05 HS vs. Control

All data obtained using CEUS imaging are presented in Figs. 5 and 6. Peak enhancement (PE) was stable in the renal arterial system in both the HS and control experiments. However, in comparison to BL, PE was significantly increased in HS at shock, early-, and late resuscitation indicating volume depletion in the renal cortex (p < 0.05). Full Width at Half Maximum (FWHM, Fig. 6A) was unaltered in the renal arterial system in both the HS and Control groups but was lower in the renal cortex of the HS group at early and late resuscitation than at BL (p < 0.05), indicating an increase in blood velocity (Fig. 6B). During early resuscitation, FHWM was also found lower (indicating increased flow) than in the control group at the same time point (p < 0.05). In addition to the lower FWHM at T2 and T3 indicating an increased blood velocity during resuscitation, the IRMTT showed a longer transit time of microbubbles in the shock phase (T1) indicating a decrease of velocity, but shorter transit times during early resuscitation compared to the control group (p < 0.05, early resuscitation vs. T2).

### Sublingual microcirculation

**Fig. 7:**
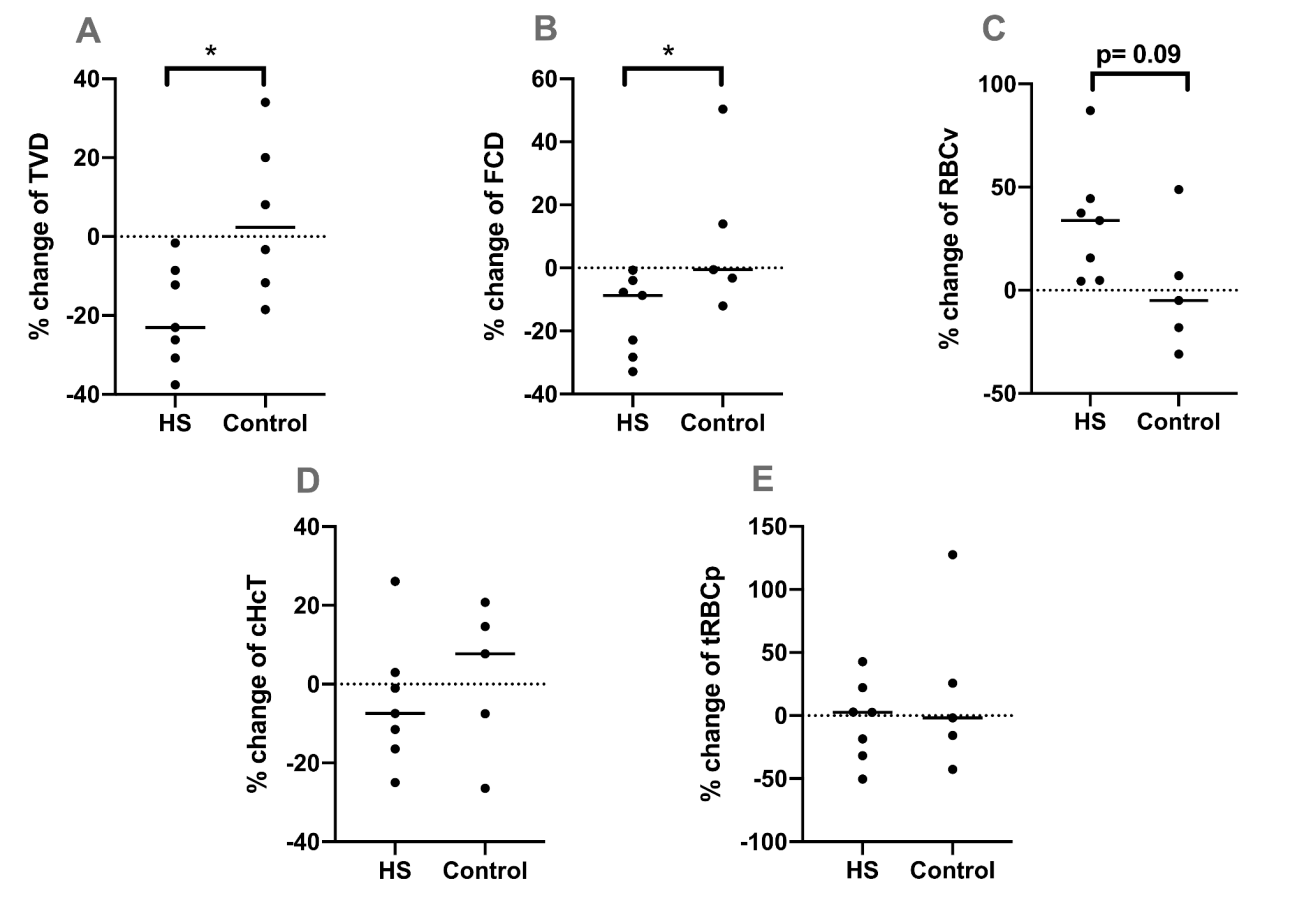
The percentage change of total vessel density (TVD), functional capillary density (FCD), red blood cell velocity (RBCv), capillary hematocrit (cHcT) and tissue red blood cell perfusion (tRBCp) from BL to T3 throughout the experiments between the HS and Control groups. Values represented as mean ± SD, * p < 0.05 HS vs. Control

The percentage change of the sublingual microcirculatory parameters in HS and BL are reported in Fig. 7. TVD has a mean decrease of 19,9% (95% CI (-31,9 − 8,06)) and FCD decreased 15% (95% CI (-26,8 − 3,2)) from BL to T3. Both parameters were significantly lower in the HS group than in the Control group (Mean TVD 4,7%; 95% CI (-16,1 25,6)) and mean FCD 9,6; 95% CI (-20,8 40,2) (p < 0.05). RBC velocity (RBCv) in sublingual capillaries increased about 32 ± 2% with respect to Control (0,4 ± 30%) but did not reach significancy (p < 0.09). In addition, no significant differences were found in capillary hematocrit (cHcT) and tissue RBC perfusion (tRBCp) throughout the shock and resuscitation process in both the HS and Control groups.

## Discussion

In this study, we performed a detailed analysis of the renal microcirculation and perfusion during shock and fluid resuscitation in a pig model of non-traumatic hemorrhage induced AKI. The results of this study provide better insights to understanding the response of peripheral- and renal microcirculation and their relation to systemic hemodynamic variables. During shock, we demonstrated that renal perfusion and RBC velocity were prominently reduced in the renal cortex despite marginally altered RBF and urine output. A slow movement of RBCs and low perfusion in the kidney microvasculature might be causing persistency of tissue hypoxia and convection deficiency in the shock phase and it could play a role in ischemia/reperfusion injury following fluid resuscitation. Indeed, following normalization of systemic hemodynamics, renal blood flow, urine output and renal perfusion after fluid resuscitation, we demonstrated that substantially slow intra-renal blood flow increased after initiation of resuscitation (hyperemia), potentially causing further endothelial and renal damage as we showed in correlation of increased plasma sialic acid and NGAL levels. We also showed that reduced TVD and FCD in the sublingual microcirculation can also be used as an indicator of global microcirculatory status.

Herein, we showed that HS is accompanied with low MAP, CO, CVP, and high HR. Despite MAP, CO, and CVP were corrected by the balanced crystalloid resuscitation, both SVR and PAP were impaired during resuscitation due to the lower viscosity of the blood and lower shear stress. Moreover, HR also remained high following resuscitation because of the physiological compensatory response to maintain CO and DO_2_ during hemodilution. Due to the reduction of DO_2_, an increased oxygen requirement caused an increase in ERO_2_ during the shock phase, but both were restored following initiation of fluid resuscitation. Therefore, we suggested that the systemic vasodilatation, low blood viscosity, and low systemic vascular resistance might contribute to the systemic and renal perfusion defects. In this study, we also showed that aggressive fluid resuscitation itself could restore the systemic variables and acid-base status. However, high lactate and creatinine levels indicated that tissue hypoxia and renal injury even persisted after fluid resuscitation.

The hemodynamic coherence between macro- and microcirculation is a corner stone to improve organ perfusion and oxygenation in the systemic hemodynamic driven by the fluid resuscitation strategy [24]. We demonstrated that fluid resuscitation itself is able to improve the systemic hemodynamic parameters, except for SVR. However, the sublingual microcirculation parameters such as TVD and FCD were still impaired after resuscitation with crystalloid. Komori et al. recently showed that vessel density, perfusion rate, arteriolar diameter, and even flow velocity were not improved by saline resuscitation compared to colloid in a rabbit model of resuscitated hemorrhagic shock [25]. Interestingly, despite RVR was normalized probably due to renal autoregulation, SVR was still depleted throughout the experiment because of the low blood viscosity. In our previous study, we demonstrated that high RBC velocity could persist during hemodilution with HES, which resulted in renal damage without the association of hyperlactatemia despite sustained or, in some part, improved systemic and renal hemodynamics [26]. However, in the present study, we found that the sublingual RBC velocity was not influenced after normalization of systemic hemodynamics by crystalloid resuscitation, but there were less capillaries than at baseline measured by a decrease of TVD and FCD. This might be the reason of high lactate levels during HS and resuscitation. Therefore, we concluded that improvement of the systemic hemodynamics might not always result in parallel improvement of the renal and sublingual microcirculatory parameters in hemorrhagic shock and resuscitation. At the end, the changes of sublingual functional capillary density reflect the global microcirculation and perfusion defect due to persistent hyperlactatemia in this model of shock.

In the kidney, RBF, RVR, creatinine clearances, and urine output improved after fluid resuscitation. These results confirm that fluid resuscitation itself provides adequate intravascular volume expansion to maintain renal hemodynamic and renal function. However, despite the CEUS PE had not changed in the renal arterial system, it was elevated in the renal cortex during shock and resuscitation indicating volume depletion in the renal cortex even after normalization of both renal and systemic hemodynamics. Wu et al., demonstrated that fluid resuscitation was able to improve cortical microcirculatory blood flow assessed by LSI in a rat model of hemorrhagic shock and resuscitation [27]. In the present study, LSI measurements also revealed that cortical perfusion was depleted in shock phase, but normalized after fluid resuscitation. The intra-renal volume discrepancy between CEUS-derived PE and LSI can be explained by high RBC velocity measured by LSI that might cover up the volume reduction in the renal cortex, which is technically able to detect moving particles. Indeed, despite unaltered RBC velocity in the renal arterial system, high flow velocity (lower FWHM) was measured in the renal cortex, especially in the early resuscitation phase. Another important parameter in this study is the IRMTT which is a measure for the movement of RBC from the renal artery to the renal cortex during shock. The IRMTT showed faster flow during early resuscitation, which is consistent with the high flow velocity following the FWHM results. This might be the reason of the low renal vascular resistance (RVR) and intra-renal vascular volume depletion during the shock phase. Moreover, we also showed that at the end of resuscitation, serum creatinine levels were still in the range of definition of AKI. Increased NGAL and sialic acid levels revealed the persistence of renal injury and endothelial matrix degradation. It was recently found that plasma NGAL seems to be an early identification marker of AKI rather than the creatinine and urea levels [28]. On the other hand, sialic acid is mostly seen in the podocytes cell membrane in glomeruli and is responsible for protecting the cell from complement mediated autolytic attack [29–31]. Damage in podocytes membranes may account for an altered glomerular filtration and glomerular damage after fluid resuscitation. Finally, we concluded that intra-renal and sublingual microcirculatory response during shock and resuscitation might show similar trends in terms of perfusion defect, volume depletion, and RBC velocity. However, Wu et al., pointed out that microcirculatory response of different splanchnic organs such as liver, kidney, intestine, and muscle may vary during fluid resuscitation [27].

In this study, we showed that aggressive fluid resuscitation could improve the systemic hemodynamic parameters such as MAP, CVP, and cardiac output following non-traumatic hemorrhagic shock, despite low systemic vascular resistance (SVR) and consistent tissue hypoxia shown by plasma lactate and serum creatinine levels. Similar to our previous study in stepwise hemodilution in pigs [26], it is shown that the correction of systemic hemodynamic variables was not accompanied with the improvement of renal cortical perfusion, intra-renal blood volume, renal damage, and systemic microcirculation following 1 h of fluid resuscitation. Lastly, we suggest that, regardless of fluid type, fluids need to be administered to patients with caution, and the measurement of renal injury biomarkers and microcirculatory assessments can be used for guiding the optimization of therapies.

### Limitations

In this study, we have some limitations that should be considered for further studies: (1) the duration of resuscitation was too short to assess the long-term effects of fluid on systemic hemodynamics, renal perfusion and function, (2) the measurements of renal medullary perfusion are lacking, (3) no histological assessment of the kidney was performed to show possible tubular and glomerular damage.
